# A national survey of burnout amongst Canadian Royal College of Physicians and Surgeons of Canada emergency medicine residents

**DOI:** 10.36834/cmej.68602

**Published:** 2020-09-23

**Authors:** Robin Liu, Kristine Van Aarsen, Rob Sedran, Rodrick Lim

**Affiliations:** 1 Schulich School of Medicine and Dentistry, Western University, Ontario, Canada; 2 Division of Emergency Medicine, Department of Medicine, Western University, Ontario, Canada; 3 Department of Paediatrics, University of Western Ontario, Ontario, Canada

## Abstract

**Background:**

In recent years, there has been growing interest in the field of physician wellness and burnout. The prevalence of burnout is non-uniform between medical specialties and is most prevalent amongst emergency medicine physicians. Importantly, burnout can be observed amongst individuals early in their medical careers, including medical students and residents. Despite ample studies in other populations, there is no national perspective of burnout amongst Canadian Royal College of Physicians and Surgeons of Canada (RCPSC)Emergency Medicine (EM) residents.

**Methods:**

Our study surveyed Canadian residents undergoing EM training though the RCPSC via local program directors using an anonymous electronic form. Basic demographic characteristics and residents’ contemplation of suicide were surveyed. The Maslach Burnout Inventory – Human Services Survey (MBI-HSS) for medical personnel was used to assess burnout on three dimensions (emotional exhaustion, depersonalization and personal accomplishment).

**Results:**

A total of 65 valid responses were collected from eight of 14 eligible institutions (response rate = 30%). Respondents are primarily male (58%) and in their postgraduate year (PGY) 1-3 (71%). Overall, 62% of residents met the threshold for burnout according to a widely cited definition of burnout using the MBI-HSS. Additionally, 14% contemplated suicide during their training. There was no statistical significance in burnout rates between male and female responders or between residents in different stages of training.

**Conclusion:**

Our results suggest significant burnout amongst Canadian EM residents. These results point to an important opportunity to better support EM residents during their training to improve wellness and reduce burnout.

## Abstract (fr)

**Contexte:**

Ces dernières années, le bien-être et l’épuisement professionnel des médecins suscitent de plus en plus d’intérêt. La prévalence de l’épuisement professionnel n’est pas uniforme ente les spécialités médicales et est plus marquée chez les urgentologues. On peut observer l’épuisement professionnel tôt en carrière , incluant chez les étudiants en médecine et résidents. Malgré les études approfondies menées auprès d’autres populations, il n’existe aucune perspective nationale de l’épuisement professionnel chez les résidents en médecine d’urgence du Collège royal des médecins et chirurgiens du Canada (CRMCC).

**Méthodes:**

À l’aide d’un formulaire électronique anonyme, nous avons sondé les résidents canadiens des programmes du CRMCC enen médecine d’urgence par le biais des directeurs de programme. Le sondage a examiné les caractéristiques démographiques de base et les idées suicidaires chez les résidents. Nous nous sommes servis de Maslach Burnout Inventory – Human Services Survey (MBI-HSS) (MBI-HSS) à l’intention du personnel médical pour évaluer l’épuisement professionnel sur trois plans (épuisement émotionnel, dépersonnalisation et réalisation personnelle).

**Résultats:**

Nous avons recueilli 65 réponses valides de huit des 14 établissements admissibles (taux de réponse = 30%). Les répondants sont majoritairement des hommes (58%) qui font des études postdoctorales (R1 àR3 (71%). En général, 62% des résidents ont atteint le seuil d’épuisement selon une définition de l’épuisement couramment utilisée dans le MBI-HSS. De plus, 14% ont envisagé de se suicider durant leur formation. Les taux d’épuisement des hommes et ceux des femmes sondées ne présentaient pas de différence statistiquement significative, tout comme ceux entre les résidents à diverses étapes de leur formation.

**Conclusion:**

Nos résultats indiquent que l’épuisement professionnel est important chez les résidents en médecine d’urgence canadiens. Par conséquent, ils fournissent un excellent point de départ pour mieux soutenir ces résidents au cours de leur formation afin d’améliorer leur mieux-être et réduire leur épuisement professionnel.

## Introduction

The issue of burnout in physicians has drawn tremendous attention by many national organizations as a critical issue in healthcare.^1^^,^^2^^,^^3^ A recent study by the Canadian Medical Association has shown that one in four of all physicians are currently suffering from burnout. Burnout has been shown to negatively impact physician mental health and patient care and safety. Moreover, it has been linked to substance use in physicians and early retirement from professional practice.^2^^,^^4^^,^^5^^,^^6^^,^^7^ The relevance of burnout to emergency medicine (EM) practice is predominant, since burnout has consistently been shown to be highest amongst EM physicians.^8^^.^^9^

Burnout has been shown to impact not only staff physicians but residents as well.^10^ Some studies suggest that burnout may be greatest during and shortly after completion of training.^10^ A study of EM residents from eight American training programs has shown that 76.1% of residents were experiencing burnout during their training.^11^ Researchers have proposed that the chaotic work environment of the emergency room, the ambiguity of clinical decisions and the high acuity of the patients all contribute to burnout among emergency medicine physicians.^12^^,^^13^

To date, there is no national data of burnout amongst RCPSC-trained EM residents. Much of our data on burnout in EM residents comes from American training programs. However, given the large differences in training structure and the culture of healthcare between the countries, it is unknown whether the prevalence of burnout in Canadian EM residents would be comparable. The purpose of our study was to determine the nature and extent of burnout in this population. Subsequent research in our Canadian population could inform the benefits of wellness strategies implemented early in an EM physician’s career.

## Methods

This study was approved by the Health Sciences Research Ethics Board at Western University. Completion of the survey was considered implied consent. In December 2018, an invitation to complete an anonymous electronic survey was e-mailed to 14 program directors at the 14 Canadian Universities with a RCPSC-regulated Emergency Medicine Residency program. Program Directors were asked to distribute the survey invitation to all residents in their training program. The survey consisted of question surrounding basic demographics (age, gender, PGY year, training site), as well as suicidal ideation during residency training. Limited demographics information was gathered to ensure that the survey data remain non-identifiable given the small size of the training programs.

The Maslach Burnout Inventory – Human Services Survey (MBI-HSS) for medical personnel was included in the survey. The MBI-HSS was used to assess burnout on three dimensions (emotional exhaustion, depersonalization and personal accomplishment). The MBI-HSS was scored as outlined in the instruction manual.^1^^4^ Burnout was dichotomized as present or absent if respondents met criteria in either the EE or DP dimensions (EE > 26 or DP > 9).^11^

Embarking on research in burnout is very challenging. Different studies have used various combinations and thresholds for burnout, as there is no universally accepted definition. We attempted to choose the most common definition in the literature. A paper analyzing medical literature on burnout discovered that the majority of studies did not include a low PA to define burnout as we did in this study.^1^^5^

Survey results were summarized using proportions with percentages or means and standard deviations where appropriate. Statistical analyses comparing the proportion of participants meeting the criteria for burnout across groups were calculated using *chi*-square, fishers exact test or Fisher-Freeman-Halton Exact test performed on R (version 3.5.1).

## Results

Of the 14 training sites, eight programs distributed the survey to their residents. For participating programs, after removing 10 responses with incomplete data, there was an overall response rate of 30% (65/217). Amongst the survey participants, 58% were male, 41% were female and 1% preferred to not disclose. The distribution between training years were as follows: PGY1 (22%), PGY2 (15%), PGY3 (27%), PGY4 (19%) and PGY5 (17%). A comparison was undertaken to compare our survey-responder sample with residents across all 14 training sites, as well as responder versus non responding sites, and no difference was found.

**Table 1:** Demographic characteristics of Canadian RCPSC of emergency medicine residents in 2018-2019

Demographic		Survey responders	Responder sites	Non-responder sites	All training sites
Gender	Male	38 (58%)	128 (59%)	108 (59%)	236 (59%)
	Female	27 (41%)	89 (41%)	74 (41%)	163 (41%)
Training Year	PGY1	14 (22%)	41 (19%)	37 (21%)	78 (20%)
	PGY2	10 (15%)	41 (19%)	34 (18%)	75 (19%)
	PGY3	18 (27%)	44 (20%)	35 (19%)	79 (20%)
	PGY4	12 (19%)	45 (20%)	38 (21%)	83 (20%)
	PGY5	11 (17%)	46 (21%)	38 (21%)	84 (21%)
Stage of Training	Junior	24 (37%)	82 (38%)	71 (39%)	153 (38%)
	Senior	41 (63%)	135 (62%)	111 (61%)	246 (62%)

Results from the MBI-HSS were scored according to the Maslach manual.^8^ The proportion of residents meeting the threshold for burnout in each MBI dimension was as follows: emotional exhaustion (35%) and depersonalization (55%) and personal accomplishment (31%). The mean scores and standard deviations in all three categories of the MBI were: Emotional Exhaustion (mean = 22.0, standard deviation = 11.3), Depersonalization (mean = 11.9, standard deviation = 6.9), Personal Accomplishment (mean = 37.1, standard deviation = 7.5).

Overall, 62% of residents met the predetermined definition of burnout (EE > 26 or DP > 9). Alarmingly, 14% reported considering suicide during their training (9/65).

**Figure 1:**
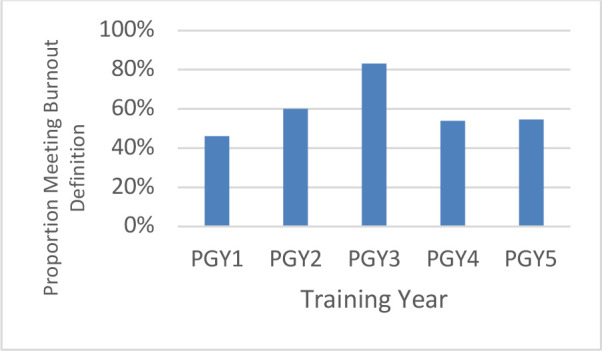
Burnout prevalence amongst residents in RCPSC emergency medicine training programs

When we explored the prevalence of burnout between demographic groups, male residents had lower burnout scores than female residents (55% versus 69% respectively), though this finding was not statistically significant (*p* = 0.261). Although burnout rates in all PGY years were high with the highest in PGY3 (83.3%) there was no statistically significant difference across years (*p* = 0.242). (Figure 1)

## Discussion

Emergency medicine has been identified as having a significantly higher percentage of burnout compared to other specialties.^8^^,^^9^ Moreover, residents and physicians starting their practice have also been identified as a high-risk group.^10^^,^^11^ Our study is an important national study that measured burnout among Canadian EM residents. The results suggest a high prevalence of burnout even among EM residents early in their professional careers. Additionally, the self-reported prevalence of suicide contemplation is alarmingly high among this population.

Senior residents appeared more likely to experience burnout, but this finding was not statistically significant. This suggests that burnout is not necessarily linked to duration of practice and that interventions to prevent burnout are as needed for residents beginning their training as those transitioning to independent practice.

We used the same definition of burnout as an American study by Lin et al.^11^ In their study of 1128 EM residents, they found a burnout prevalence of 76.1% amongst respondents.^11^ This higher prevalence of burnout in EM residents in American training programs could be attributed to the numerous differences in the healthcare system between the two countries or the demands of training programs. Nonetheless, the prevalence of burnout in our study highlights the importance for residency programs to implement strategies for fighting burnout.

For comparison purposes, we experimented with the application of more liberal definitions of burnout (EE>26 or DP>9 or PA<34) which includes a burnout threshold for PA of less than 34 in our population. This would have increased the number of participants meeting burnout criteria from 62% to 72.3% although the differences between groups remained statistically non-significant.

There have been discussions in Canada about strategies aimed at prioritizing resident wellness. Indeed, a list of 31 recommendations were made in a recent CJEM position statement on wellness in residency.^1^^6^ These recommendations included strategies such as building resiliency skills, providing residents with access to supports and improving the workplace culture around wellness. A subsequent commentary from Ting et al discussed practical considerations of implementing these strategies in a demanding training program.^1^^7^ There is some support for restricting duty hours and organized self-care workshops.^1^^8^^,^^19^^,^^20^^,^^21^

Limitations to this study include the lack of participation from six of the 14 EM programs in Canada. However, of the programs that did participate, we achieved a response rate of 30%, with an appropriate geographical distribution of the participating training sites, and demographic characteristic of residents when compared to the total Canadian EM resident population. Nonetheless, the low response rate could have introduced bias to the results based on the residents who chose to participate. Although some of our results could showed differences, none met statistical significance. Additionally, our scope was limited to providing the state of burnout among RCPSC of Emergency Medicine residents without including residents participating in the Canadian College of Family Physicians with Emergency Medicine Certification (CCFP-EM) training route. Researchers and medical education leaders should consider measuring burnout among these and all resident groups as they implement programs to mitigate burnout.

## Conclusion

Our results suggest substantial burnout amongst EM residents that is comparable to practicing staff physicians. Alarmingly, 14% of responders reported having contemplated suicide during their training. These results point to an important imperative to better support EM residents during their training to improve wellness and reduce burnout throughout their careers. Our study provides a snapshot of burnout prevalence among Canadian EM residents. With these baseline data available, we intend to investigate the efficacy of different interventions to improve resident wellness and reduce burnout among trainees.

## References

[1] World Health Organization. (2018). International classification of diseases for mortality and morbidity statistics (11^th^ Revision). Retrieved from https://icd.who.int/browse11/l-m/en [Accessed July 7, 2020].

[2] Physician burnout: A global crisis. Lancet, 2019;394(10193): 93 10.1016/S0140-6736(19)31573-9 31305255

[3] Canadian Medical Association. CMA national physician health survey (nphs) – A national snapshot. Ottawa; The Association; 2018.

[4] Lu DW, Dresden S, McCloskey C, Branzetti J, Gisondi MA Impact of burnout on self-reported patient care among emergency physicians. West J Em, 2015;16(7): 996-1001. 10.5811/westjem.2015.9.27945 PMC4703144 26759643

[5] Brown SD, Goske MJ, Johnson CM Beyond substance abuse: stress, burnout, and depression as causes of physician impairment and disruptive behaviour. J Amer Coll Rad 2009;6(7): 479-485. 10.1016/j.jacr.2008.11.029 19560063

[6] Dewa CS, Jacobs P, Thanh NX, Loong D An estimate of the cost of burnout on early retirement and reduction in clinical hours of practicing physicians in Canada. BMC Health Serv Res 2014;14: 254 10.1186/1472-6963-14-254 24927847 PMC4062768

[7] Wallace JE, Lemaire JB, Ghali WA Physician wellness: a missing quality indicator. The Lancet 2009;374(9702): 1714-1721. 10.1016/S0140-6736(09)61424-0 19914516

[8] Shanafelt TD, Boone S, Tan L, et al Burnout and satisfaction with work-life balance among US physicians relative to the general US population. JAMA 2012;172(18): 1377-1385. 10.1001/archinternmed.2012.3199 22911330

[9] Shanafelt TD, West CP, Sinsky C, et al Changes in burnout and satisfaction with work-life integration in physicians and the general US working population between 2011 and 2017. Mayo Clinic Proceedings 2019;94(9): 1681-1694. 10.1016/j.mayocp.2018.10.023 30803733

[10] Rodrigues H, Cobucci R, Oliveira A, et al Burnout syndrome among medical residents: A systematic review and meta-analysis. Plos One 2018;13(11): e206840 10.1371/journal.pone.0206840 PMC6231624 30418984

[11] Lin M, Battaglioli N, Melamed M, Mott SE, Chung AS, Robinson D High prevalence of burnout among US emergency medicine residents: results from the 2017 National Emergency Medicine Wellness Survey. Ann Emerg Med 2019;74(5): 682-690. 10.1016/j.annemergmed.2019.01.037 30879701

[12] Takayesu JK, Ramoska EA, Clark TR, et al Factors associated with burnout during emergency medicine residency. Soc Acad Emerg Med 2014;21: 1031-1035. 10.1111/acem.12464 25269584

[13] Stehman C, Testo Z, Gershaw R, Kellogg A Burnout, Drop Out, Suicide: Physician Loss in Emergency Medicine, Part I. West J Emerg Med. 2019 5; 20(3): 485–494. 10.5811/westjem.2019.4.40970 31123550 PMC6526882

[14] Maslach C, Schaufeli WB, Leiter MP Job burnout. Annu Rev Psychol 2001;52:397-422. 10.1146/annurev.psych.52.1.397 11148311

[15] Doulougeri K, Georganta K & Montgomery A Diagnosing burnout among healthcare professionals: Can we find consensus?. Cogent Medicine. 2006;3(1). 10.1080/2331205X.2016.1237605

[16] Taher A, Crawford S, Koczerginski J, et al Position statement on resident wellness. CJEM 2018;0(0):1-14. 10.1017/cem.2018.8

[17] Ting DK, Baylis J From abstraction to action: Making wellness practical during residency training. CJEM 2018;20(5): 662-664. 10.1017/cem.2018.445 30205862

[18] Goitein L, Shanafelt TD, Wipf JE, Slatore CG, Back AL The effects of work-hour limitations on resident well-being, patient care, and education in an internal medicine residency program. Arch Intern Med 2005;165(22): 2601-2606. 10.1001/archinte.165.22.2601 16344417

[19] Gopal R, Glasheen JJ, Miyoshi TJ Burnout and internal medicine resident work-hour restrictions. Arch Intern Med 2005;165(22): 2595-2600. 10.1001/archinte.165.22.2595 16344416

[20] Martins AE, Davenport MC, de la Paz Del Valle M, et al Impact of a brief intervention on the burnout levels of pediatric residents. J Pediatr 2011;87(6): 493-498. 10.2223/JPED.2127 22170452

[21] Busireddy KR, Miller JA, Ellison K, Ren V, Qayyum R, Panda M Efficacy of interventions to reduce resident physician burnout: A systematic review. J *Grad Med Educ* 2017;9(3): 294-301. 10.4300/JGME-D-16-00372.1 28638506 PMC5476377

